# Risk factors of preoperative myocardial injury in patients with gastrointestinal tumors

**DOI:** 10.1186/s12872-023-03086-1

**Published:** 2023-02-25

**Authors:** Shuqi Yu, Shiyao Cheng, Jinhong Si, Huajing Peng, Jiachen Wan, Jiaojie Xue, Zhichong Chen, Sutian Hu, Ling Zhou, Yitao Zhang, Weijie Zeng

**Affiliations:** 1 Department of Cardiovascular Internal Medicine, The Sixth Affiliated Hospital of Sun Yat-Sen University, Guangzhou, 510655 China; 2 Department of Respiratory, The Affiliated Hexian Memorial Hospital of Southern Medical University, Guangzhou, 511400 China; 3 Department of Renal Internal Medicine, The First Affiliated Hospital of Sun Yat-Sen University, Guangzhou, 510000 China; 4 Tsinghua Shenzhen International Graduate School, Tsinghua University, Shenzhen, 518055 China; 5 Hospital of South, China University of Technology, Guangzhou, 510000 China

**Keywords:** Gastrointestinal tumor, Myocardial injury, High-sensitive cardiac troponin I, Risk factor

## Abstract

**Background:**

Recent studies indicated that the prognosis of patients with gastrointestinal tumors is frequently influenced by its complications, notably myocardial injury. The main object is to investigate the occurrence and risk factors of myocardial injury in patients with gastrointestinal tumor.

**Methods:**

1126 patients who received gastrointestinal tumor related surgery from May 2018 to June 2020 in the Sixth Affiliated Hospital of Sun Yat-sen University were retrospectively collected and divided into the non-myocardial injury group and the myocardial injury group (high-sensitive cardiac troponin I (hs-cTnI) ≥ 0.028 ng/ml). The occurrence and risk factors of myocardial injury in patients with gastrointestinal tumor are analyzed. The influence of myocardial injury on the ICU detention time in gastrointestinal tumor patients is also studied.

**Results:**

In total, 78 (6.93%) patients developed myocardial injuries. Compared with patients in the non-myocardial injury group, patients in the myocardial injury group have a higher prevalence of cardiovascular risk factors (including advanced age and higher smoking ratio), a higher prevalence of comorbidities (such as previous coronary artery disease, hypertension, atrium fibrillation and diabetes), and a higher rate of premedication (such as anticoagulation, β-blocker, Angiotensin-converting enzyme inhibitor/Angiotensin II receptor blocker, and diuretic) (all with *P*-value < 0.05). In addition, patients in the myocardial injury group also presented with a higher revised cardiac risk index (Lee index), higher neutrophil granulocyte ratio, lower hemoglobin, and higher likelihood of impaired cardiac structure and function (all with *P*-value < 0.05). There was a trend of statistical significance in the ICU detention time between the myocardial injury group and the non-myocardial injury group (1[1,3] vs. 2[1,10], *P* = 0.064). In this study, there were 7 patients presented with clinical symptoms in the myocardial injury group (chest discomfort in 4 cases, non-compressive precordial chest pain in 1 case, dyspnea in 2 cases). In the multivariate analysis, advanced age, increased Lee index score, increased neutrophil granulocyte ratio, decreased left ventricular ejection fraction (LVEF), increased interventricular septum were independent risk factors for myocardial injury.

**Conclusion:**

In conclusion, advanced age, increased Lee index, increased neutrophil granulocyte ratio, decreased left ventricular ejection fraction, and increased ventricular septum were independent risk factors for preoperative myocardial injury in patients with gastrointestinal tumors. The proportion of clinical symptoms in gastrointestinal tumor patients with myocardial injury was low, indicating the necessity to closely monitor the cardiac status of individuals with gastrointestinal tumors.

**Supplementary Information:**

The online version contains supplementary material available at 10.1186/s12872-023-03086-1.

## Introduction

Gastrointestinal tumor has become one of the most prevalent tumors around the world, affecting millions of people worldwide [1, 2]. Early diagnosis and development in tumor treatment have greatly improved gastrointestinal tumor patients’ overall survival rate. However, these patients often die of cardiovascular disease rather than recurrence of their tumor [3].

Hs-cTnI (high-sensitive cardiac troponin I) is a index with high specificity and sensitivity for the detection of myocardial injury, which can reflect the myocardial injury of non-cardiogenic diseases. Elevated levels of hs-cTnI is associated with higher mortality in non-cardiac hospitalized patients [4–6], including patients with gastrointestinal tumors [7]. Taken together, myocardial injury should be given high priority [8]. Considering the shared cardiovascular risks between tumors and cardiovascular disease [9], inflammatory states associated with malignancies, and cardiotoxic effects of cancer therapy, gastrointestinal tumors complicated with myocardial injury may be common. Most studies focus on the monitoring of myocardial injury during the treatment of gastrointestinal tumors, ignoring the possible myocardial injury caused by the disease itself. It may be of great significance to clarify risk factors of myocardial injury before surgery for the necessity of taking preventive intervention. Furthermore, preoperative intervention of myocardial injury may bring a better prognosis to patients.

However, to the best of our knowledge, no studies have reported the incidence and risk factors of myocardial injury in patients with gastrointestinal tumors. This is a retrospective study to investigate the risk factors leading to myocardial injury in patients and thus screening out patients with high-risk gastrointestinal tumors complicated with myocardial injury.

## Methods

### Study design and participants

**Fig. 1:**
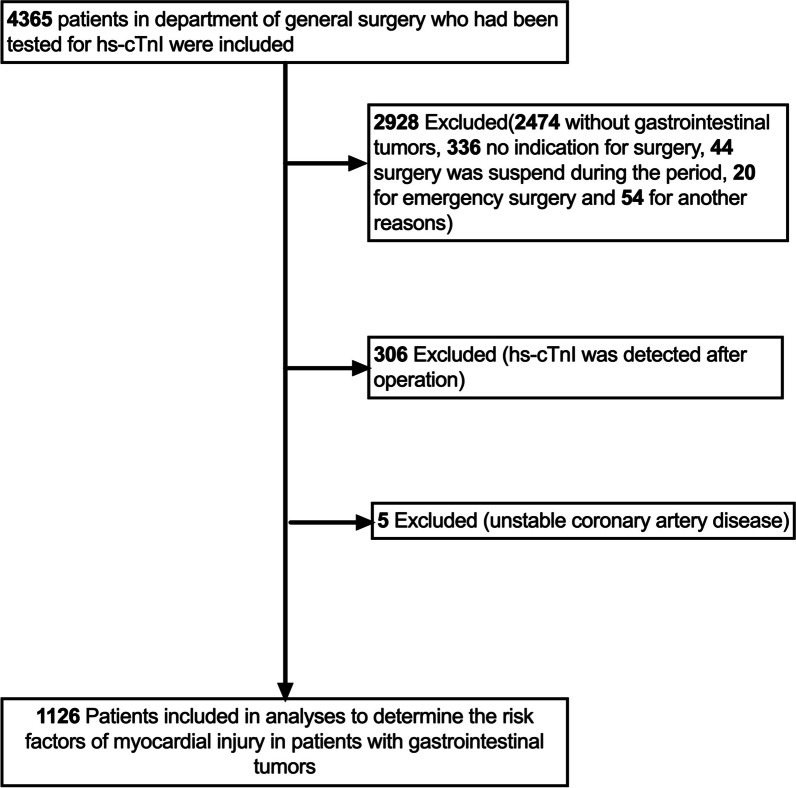
Flow diagram that shows the process of enrollment and exclusion. Hs-cTnI, high sensitive-cardiac troponin I

This was a single-center retrospective study. Patients who admitted to the Sixth Affiliated Hospital of Sun Yat-sen University from May 2018 to June 2020 and had been tested for hs-cTnI were screened. 1126 consecutive patients who compliance with inclusion criteria and were finally enrolled (Fig. 1).

Patients with gastrointestinal tumors (age ≥ 18 years old) who underwent tumor related surgery under general anesthesia and completed hs-cTnI testing at admission within 7 days before surgery were consecutively enrolled. Exclusion criteria were emergency surgery, failure to received tumor related surgery, and clinical evidence of acute coronary syndrome (typical angina pectoris with or without ST-segment elevation electrocardiograph alteration) in the medical history of preoperative assessment. The database of this study comes from the study published in EClinicalMedicine in August 2021 [7].

This study has been approved by the Medical Ethics Committee of the Sixth Affiliated Hospital of Sun Yat-sen University (2021ZSLYEC-296).

### Study definitions

History of coronary heart disease, defined as undergoing previous bypass surgery, coronary intervention, myocardial infarction, or meeting the guidelines definition [10, 11]. Myocardial infarction was diagnosed using the universal definition of myocardial infarction [12].

Lee index (revised cardiac index) was calculated as follows: high-risk surgical type, history of ischemic heart disease, history of congestive heart failure, history of cerebrovascular disease, history of insulin therapy for diabetes mellitus, and preoperative serum creatinine > 2.0 mg/dL (176.8 μmol/L) accounting for 1 point each [13].

### Data collection

We analyzed the data of the most recent examination before surgery during hospitalization. Data including patients’ characteristics, laboratory index and echocardiographic parameters at baseline, comorbidities, premedication and outcome (the ICU detention time) were all collected from medical records. Patients were divided into the myocardial injury group and the non-myocardial injury group according to the hs-cTnI elevation (≥ 0.028 ng/ml) at admission.

Blood samples were collected within 7 days before surgery. The samples were collected from the antecubital vein, stored in additive-free test tubes, and processed immediately. Using centrifugation to separate serum and stored it at -70℃ until analysis. On an analyzer, hs-cTnI was quantified using a high-sensitivity electrochemiluminescence-immunoassay (ABBOTT, Architect i1000SR). The 99th percentile reference value of hs-cTnI was 0·028 ng/ml. All of the tests were carried out at Sun Yat-sen University’s Sixth Affiliated Hospital's clinical laboratory.

All echocardiographic examinations were conducted during hospitalization prior to surgery. The images were recorded using a digital ultrasound device and archived according to the requirements of the American Society of Echocardiography (GE vivid E9).

### Statistical analysis

We used SPSS22.0 statistical software to process all analyses. Continuous variables are expressed as means ± standard deviation or median [25%, 75%], and classification variables are expressed as frequency (percentage). Continuous variables were compared using the independent T-test or paired sample T test or Mann–Whitney U test, and categorical variables were compared using the Chi-square test or Fisher's exact test where appropriate. Odds ratios for candidate variables were calculated by univariate logistic regression analyses. The indexes with significant differences were analyzed by multivariate logistic regression, and a *P*-value < 0.05 was considered statistically significant. Missing values were excluded from the relevant analysis.

## Results

### Baseline characteristics in the myocardial injury group and non-myocardial injury group

The final sample size consisted of 1126 patients (Fig. 1). Of the 1126 patients (35.8% females; mean age 62.6 years) enrolled in this study, 78 (6.93%) patients had elevated hs-cTnI (Hs-cTnI ≥ 0.028), which is defined as myocardial injury group. The main surgical sites included: gastric (109, 9.7%), colorectum (994, 88.3%), intestinal (20, 1.8%), esophageal (1, 0.1%) and anal tube (2, 0.2%). In this study, there were 7 patients presented with clinical symptoms in the myocardial injury group (chest discomfort in 4 cases, non-compressive precordial chest pain in 1 case, dyspnea in 2 cases).

As shown in Table 1 and 2, the mean age of the 78 gastrointestinal tumor patients with myocardial injury was 71.0 ± 11.1 years, which is higher than that in non-myocardial injury group (*P* < 0.001). Compared with patients in the non-myocardial injury group, patients in the myocardial injury group have a higher prevalence of cardiovascular risk factors (including senior age and higher proportion of smoking), a higher prevalence of comorbidities (such as previous coronary artery disease, hypertension, atrium fibrillation and diabetes, and a higher rate of premedication (such as anticoagulation, β-blocker, Angiotensin-converting enzyme inhibitor/Angiotens in II receptor blocker, and diuretic) (all with *P*-value < 0.05). As compared with non-myocardial injury group, patients in the myocardial injury group also presented with a higher revised cardiac risk index (Lee index), higher neutrophil granulocyte ratio, lower hemoglobin, and higher likelihood of impaired cardiac structure and function (all with *P*-value < 0.05). In addition, 6.4% of patients from the myocardial injury group take statins, which is relatively higher than that in non-myocardial injury group. There was no statistical difference in the proportion of receiving preoperative chemotherapy between the two groups. (The chemotherapy regimen was provided in Additional file 1).

**Table 1:** Baseline characteristics

	All	All patients	n1	Non-myocardial injury group (n = 1048)	n2	myocardial injury group (n = 78)	P*
Gender (%)		723(64.2%)		667(63.6%)		56(71.8%)	0.147
Age (years)	1126	62.6 ± 11.9	1048	62.0 ± 11.7	78	71.0 ± 11.1	< 0.001
BMI (kg/m_2_)	1109	23.4 ± 3.4	1034	22.4 ± 3.3	75	22.3 ± 3.8	0.748
History							
Smoking	1126						0.003
Never smoking		996(88.5%)		928(88.5%)		68(87.2%)	
Used to smoke		115(10.2%)		110(10.5%)		5(6.4%)	
Current smoking		15(1.3%)		10(1.0%)		5(6.4%)	
CAD		85(7.5%)		65(6.2%)		20(25%)	< 0.001
PCI history		33(2.9%)		27(2.6%)		6(7.7%)	0.025
CABG history		1(0.1%)		0		1(1.3%)	0.069
Hypertension		248(22.0%)		215(20.5%)		33(42.3%)	< 0.001
Atrial fibrillation		11(0.9%)		7(0.7%)		4(5.1%)	0.005
Diabetes mellitus		113(10.0%)		98(9.4%)		15(19.2%)	0.005
Insulin dependent		17(1.5%)		11(1.0%)		6(7.7%)	< 0.001
Chronic renal failure		6(0.5%)		5(0.5%)		1(0.3%)	0.351
Dialysis		1(0.1%)		0		1(1.3%)	0.069
Medical treatment	1126						
Aspirin		24(2.1%)		21(2.0%)		3(3.8%)	0.496
Clopidogrel		29(2.6%)		25(2.4%)		4(5.1%)	0.269
Anticoagulation		22(2.0%)		15(1.4%)		7(9.0%)	< 0.001
β-blocker		34(3.0%)		27(2.6%)		7(9.0%)	0.004
ACE inhibitor/ARB		75(6.7%)		64(6.1%)		11(14.1%)	0.006
ARNI		2(0.2%)		2(0.2%)		0	1
CCB		110(9.8%)		98(9.4%)		12(15.4%)	0.083
Statins		40(3.6%)		35(3.3%)		5(6.4%)	0.273
Diuretic		20(1.8%)		15(1.4%)		5(6.4%)	0.006
Metformin		41(3.6%)		35(3.3%)		6(7.7%)	0.096
Chemotherapy		148(13.1%)		136(13.0%)		12(15.4%)	0.544
Radiation therapy		21(1.9%)		21(2.0%)		0	0.408
LEE Index	1126						< 0.001
0		993(88.2%)		943(90.0%)		50(64.1%)	
1		112(9.9%)		90(8.6%)		22(28.2%)	
≥ 2		21(1.9%)		15(1.4%)		6(7.7%)	
NYHA class II-IV		14(1.2%)		10(1.0%)		4(5.1%)	0.007
Radical operation		1006(89.3%)		951(90.7%)		55(71.4%)	< 0.001
HR (beats per min)	1125	80.4 ± 12.9	1047	80.4 ± 13.0	78	80.1 ± 11.9	0.868
SBP (mmHg)	1124	126.1 ± 18.3	1046	125.8 ± 18.1	78	130.7 ± 20.0	0.021
DBP (mmHg)	1124	76.9 ± 10.7	1046	77.0 ± 10.7	78	75.4 ± 10.4	0.184

Continuous variables are expressed as means ± standard deviation. Categorical variables are expressed as frequencies (percentage)

BMI, body mass index; CAD, coronary artery disease; ACEI, angiotensin-converting enzyme inhibitor; ARB, angiotensin receptor blocker; CCB, calcium channel blocker; ANRI, angiotensin receptor neurolysin inhibitors; NYHA, New York Heart Association; HR, heart rate; SBP, systolic blood pressure; DBP, diastolic blood pressure

*Significance of the differences between the patients with and without myocardial injury

**Table 2:** Laboratory index, echocardiographic parameters on admission

	All	All patients	n1	Non-myocardial injury group (n = 1048)	n2	myocardial injury group (n = 78)	*P*
HB (g/L)	1124	119.2 ± 24.8	1046	119.7 ± 24.9	78	111.9 ± 22.7	0.007
RDW (%)	1121	15.3 ± 5.7	1043	15.3 ± 5.8	78	15.8 ± 3.9	0.44
PDW (%)	1105	11.7 ± 2.5	1027	11.7 ± 2.5	78	12.1 ± 2.9	0.157
WBC(× 10^9/L)	1124	6.6 ± 3.2	1046	6.5 ± 3.2	78	6.9 ± 3.0	0.318
NEUR (%)	1124	29.9 ± 30.8	1046	28.3 ± 30.5	78	50.7 ± 27.1	< 0.001
PCT (ng/ml)	138	0.05 ± 0.62	136	0.05 ± 0.6	2	0.06 ± 0.1	0.989
CRP (mg/L)	105	5.5 ± 18.2	1001	5.6 ± 18.0	49	3.6 ± 21.8	0.453
Cr (μmol/L)	1115	78.2 ± 41.7	1038	76.8 ± 24.2	77	97.9 ± 130.6	0.161
LDL-C (mmol/L)	1078	3.2 ± 0.9	1006	3.2 ± 0.9	72	2.8 ± 0.7	< 0.001
AST (U/L)	1119	22.1 ± 13.5	1042	21.8 ± 13.4	77	25.4 ± 14.5	0.041
ALT (U/L)	1119	18.9 ± 17.8	1042	18.9 ± 18.1	77	18.3 ± 13.4	0.779
TBIL (g/L)	1101	12.4 ± 8.1	1025	12.4 ± 8.2	76	12.1 ± 5.6	0.736
DBIL (g/L)	1101	2.7 ± 4.2	1025	2.7 ± 4.3	76	3.2 ± 2.9	0.336
CKMB (U/L)	1034	16.5 ± 23.4	961	16.8 ± 24.2	73	13.3 ± 6.6	0.002
BNP (pg/ml)	86	204.7 ± 474.7	63	157.5 ± 434.0	23	334.1 ± 562.2	0.127
MYO (ng/ml)	1126	44.5 ± 75.9	1048	40.8 ± 40.2	78	94.2 ± 244.2	0.058
D-dimer (mg/L)	630	0.9 ± 1.5	581	0.9 ± 1.3	49	1.7 ± 2.9	0.066
CEA (ng/ml)	1107	31.8 ± 302.9	1032	31.3 ± 310.6	75	38.2 ± 164.0	0.848
CA125 (U/L)	1111	23.6 ± 70.0	1036	19.6 ± 34.3	75	79.2 ± 231.7	0.029
CA199 (U/L)	1101	243.6 ± 3814.0	1026	141.7 ± 1266.9	75	1637.7 ± 13,852.4	0.353
LVEF (%)	1015	66.6 ± 6.2	942	68.8 ± 5.9	73	63.4 ± 8.1	< 0.001
LVDd (mm)	1015	44.2 ± 5.5	942	44.1 ± 5.4	73	45.9 ± 6.0	0.006
LA (mm)	1015	30.5 ± 4.7	942	30.3 ± 4.5	73	33.1 ± 5.7	< 0.001
IVS (mm)	1015	9.5 ± 1.6	942	9.4 ± 1.5	73	10.4 ± 1.9	< 0.001
LVPW (mm)	1015	9.2 ± 1.4	942	9.1 ± 1.3	73	9.9 ± 1.7	< 0.001

Data are expressed as means ± standard deviation

HB, hemoglobin; WBC, white blood cell; NEUR, neutrophil granulocyte ratio; PCT, procalcitonin; CRP, C-reactive protein; LDL, low-density lipoprotein; ALT, alanine aminotransferase; AST, aspartate transaminase; TBIL, total bilirubin; DBIL, direct bilirubin; CKMB, creatine phosphokinase-MB; BNP, brain natriuretic peptide; MYO, myoglobin; CEA, carcinoembryonic antigen; CA125, carbohydrate antigen 125; CA199, carbohydrate antigen 199; LVEF, left ventricular ejection fraction; LVDd, left ventricular end-diastolic diameter; LA, left atrium; IVS, interventricular septum; LVPW, left ventricular posterior wall. P < 0.001

### The influence of myocardial injury on the outcome of patients with gastrointestinal tumor

Moreover, there was a statistical significance in ICU admission between the myocardial injury group and the non-myocardial injury group (22.1% vs. 4.2%, *P* < 0.001), and there was a trend of statistical significance in the ICU detention time (1[1, 3] vs. 2[1, 10], *P* = 0.064) between the two groups.

### Risk factors for myocardial injury in patients with gastrointestinal tumor

As shown in Table 3, univariate analysis revealed that advanced age, higher Lee index, increased systolic blood pressure, increased neutrophil granulocyte ratio, decreased hemoglobin, increased aspartate transaminase, higher CA125, decreased LVEF, increased left ventricular end-diastolic diameter, increased left atrium diameter, as well as increased interventricular septum thickness and left ventricular posterior wall thickness were risk factors for myocardial injury in patients with gastrointestinal tumor. Preoperative chemotherapy is not a significant risk factor in preoperative myocardial injury in patients with gastrointestinal tumors (OR = 1.219 [0.642–2.314], *P* = 0.544).

**Table 3:** Univariate logistic regression analysis of risk factors for myocardial injury

	OR	95%CI	*P*
Age (years)	1.079	1.054–1.105	< 0.001
LEE Index	3.384	2.303–4.973	< 0.001
SBP (mmHg)	1.014	1.002–1.027	0.021
HB (g/L)	0.988	0.979–0.997	0.008
NEUR (%)	1.025	1.017–1.034	< 0.001
AST (U/L)	1.012	1.000–1.024	0.043
CA-125	1.008	1.004–1.012	< 0.001
LVEF (%)	0.927	0.897–0.959	< 0.001
LVDd (mm)	1.065	1.019–1.114	0.006
LA (mm)	1.123	1.072–1.177	< 0.001
IVS (mm)	1.415	1.232–1.625	< 0.001
LVPW (mm)	1.524	1.293–1.797	< 0.001

As shown in Table 4, advanced age, increased Lee index score, increased neutrophil granulocyte ratio, decreased LVEF and increased interventricular septum were still independent risk factors for myocardial injury in multivariate logistic regression analysis. As compared with patients with Lee index = 0, OR value (95% CI) in patients with Lee index = 1 and 2 were 2.559 (1.335–4.908, *P* = 0.005) and 2.378 (0.686–8.238,* P* = 0.172), respectively.

**Table 4:** Multivariate logistic regression analysis of risk factors for myocardial injury

	OR	95%CI	*P*
Age (years)	1.065	1.036–1.096	< 0.001
NEUR (%)	1.025	1.015–1.035	< 0.001
LVEF (%)	0.936	0.901–0.972	< 0.001
IVS (mm)	1.401	1.194–1.644	< 0.001
LEE Index			0.012
LEE1vs0	2.559	1.335–4.908	0.005

## Discussion

We found in the present study 78 cases (6.93%) of 1126 patients with gastrointestinal tumors were complicated with myocardial injury. Advanced age, increased Lee index score, elevated neutrophil granulocyte ratio, decreased LVEF, and increased ventricular septum thickening were identified as independent risk factors for gastrointestinal tumor complicated with myocardial injury.

Previous studies reported myocardial injury occurs frequently (42/586, 7.17%) in patients undergoing colorectal tumor surgery in an enhanced recovery after surgery protocol [6]. We reported incidence rate of gastrointestinal tumor combined with myocardial injury before surgery, which is 6.93%. The interpretation of the results needs to be cautious since only the patients who received surgery were selected. High-risk patients may have been excluded. This may underestimate the incidence of myocardial injury in patients with gastrointestinal tumors. Further prospective multicenter studies are needed to confirm this speculation.

Our study indicates that advanced age is a risk factor for preoperative myocardial injury in patients with gastrointestinal tumors. This is different from previous reports which showed age is not a risk factor for upper gastrointestinal bleeding with simultaneous myocardial injury [14]. However, there was a study shown that elderly people are more likely to suffer from hypertension, diabetes, peripheral vascular disease, chronic heart failure, and renal failure [15]. Our study is consistent with a previous study which demonstrated advanced age is a major risk factor for cardiovascular disease [16]. The mechanisms probably as follows: firstly, aging reduced mitochondrial content and progressively slower stress response to ischemia, contributing to myocardial injury; secondly, autophagy may decrease in aging myocardium as a cellular protective cycling mechanism, leading to cardiac dysfunction and myocardial injury [17, 18]. Lee index was widely applied to identify patients at higher risk for perioperative complications or myocardial injury in patients undergoing non-cardiac surgery [13, 19]. Our study demonstrated that Lee index is an independent risk factor for preoperative myocardial injury in patients with gastrointestinal tumors. Our study seems to consistent with a previous study which reported that upper gastrointestinal bleeding patients with more than three cardiac risk factors comprised a high-risk group for simultaneously developing myocardial injury [14]. Tumor patients complicated with diabetes are in chronic hyperglycemic states, which can simultaneously cause microangiopathy in the cardiovascular system, leading to punctate necrosis of the myocardium. Elevated hs-cTnI in patients with chronic kidney disease may be associated with its reduced excretion rate, which causing myocardial damage due to toxin accumulation in the body. This mechanism may explain the relationship between Lee index and myocardial injury.

Inflammation is recognized as a prominent feature of tumor progression [20]. Studies have shown that about 20–40% of gastrointestinal cancer patients have systemic inflammation before surgery, which is one of the indicators of poor prognosis before surgery [21]. Systemic inflammation may promote the progression of myocardial injury during the perioperative period [22]. Our findings indicate that the neutrophil granulocyte ratio is an independent risk factor for preoperative myocardial injury in patients with gastrointestinal tumors. Feldstein et al. suggested that inflammation may be the mechanism for myocardial injury [23], which is consistent with our findings.

We found that LVEF reduction and ventricular septum thickening were independent risk factors for gastrointestinal tumors complicated with myocardial injury. Previous studies have shown that hs-cTnI concentration is related to left ventricular diastolic dysfunction as suggested by echocardiography [24]. Therefore, echocardiography indicators such as LVEF and ventricular septal thickness may reflect the severity of myocardial injury, which is in line with our research results.

We found that there was a trend of statistical significance in the ICU detention time between the myocardial injury group and the non-myocardial injury group, which is consistent with the results of Tota-Maharaj et al.’ s study [25]. The possible reasons are as follows: (1) The status of tumor patients complicated with myocardial injury was more complex, requiring additional cardiac examination, monitoring and intensive care.

There are several limitations in this study. Firstly, this study is a retrospective single-center study, and the inclusion of gastrointestinal tumor patients undergoing surgical treatment does not represent the incidence of myocardial injury in all patients with gastrointestinal tumors. Prospective multicenter research is warranted in the future. However, our research suggests that gastrointestinal tumor is sometimes complicated with myocardial injury, which cannot be ignored in clinical practice. Secondly, there was no statistical significance between Lee index = 2 and Lee index = 0, which may be due to the small sample size of gastrointestinal tumor patients with Lee index ≥ 2. Thirdly, in order to better assess the cardiac status of gastrointestinal tumor patients and take necessary intervention before surgery, our present study focuses on the risk factors for preoperative myocardial injury. Therefore, risk factors for postoperative myocardial injury were not investigated in the present study.

## Conclusions

In conclusion, advanced age, increased Lee index, increased neutrophil granulocyte ratio, decreased left ventricular ejection fraction, and enlarged ventricular septum were independent risk factors for preoperative myocardial injury in patients with gastrointestinal tumors. The proportion of clinical symptoms in gastrointestinal tumor patients with myocardial injury was low, indicating the necessity to closely monitor the cardiac status of individuals with gastrointestinal tumors in preoperative period.

## Supplementary Information

**Additional file 1**. Chemotherapy information that shows the chemotherapy regimen and proportion of patients in each group.

## Data Availability

The data underlying the results presented in this study are available from the corresponding author upon reasonable request.
