# Supplementary figures and images for: Risk factors of preoperative myocardial injury in patients with gastrointestinal tumors
Source: BMC Cardiovasc Disord. 2023 Feb 25;23:109. doi: 10.1186/s12872-023-03086-1 (PMC9960661; doi:10.1186/s12872-023-03086-1)

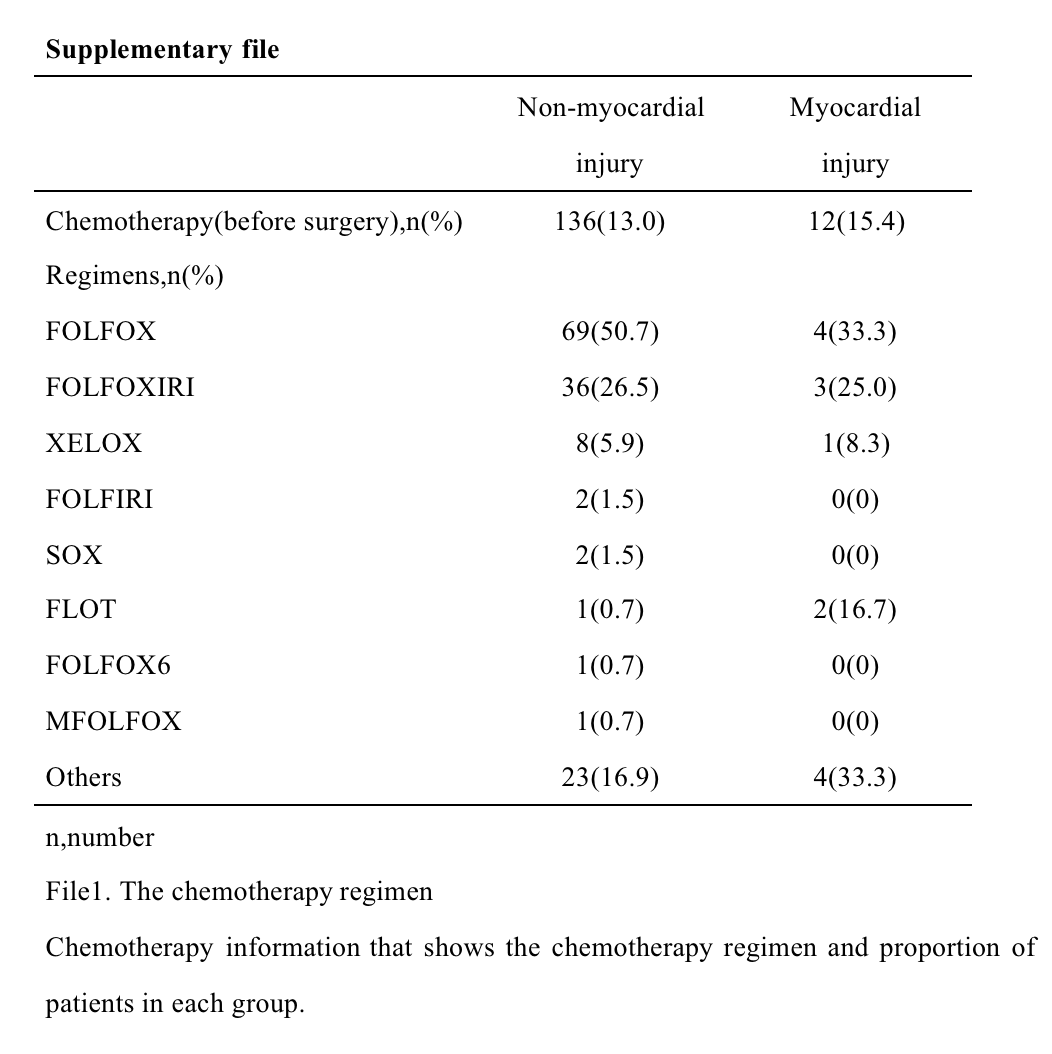

Supplement: Supplementary file 1 — Additional file 1. Chemotherapy information that shows the chemotherapy regimen and proportion of patients in each group. [file 12872_2023_3086_MOESM1_ESM.jpg]
